# 新药时代诱导化疗方案对多发性骨髓瘤自体造血干细胞动员的影响

**DOI:** 10.3760/cma.j.cn121090-20250221-00084

**Published:** 2025-12

**Authors:** 希龙 王, 萌萌 潘, 诗炜 金, 山 高, 增凯 潘, 焰 王, 怡 陶, 坚青 糜, 卫平 章

**Affiliations:** 上海交通大学医学院附属瑞金医院血液科，国家转化医学中心（上海），上海血液学研究所，医学基因组学国家重点实验室，上海 200025 Department of Hematology, Ruijin Hospital Affiliated to Shanghai Jiao Tong University School of Medicine, National Research Center for Translational Medicine at Shanghai, Shanghai Institute of Hematology, State Key Laboratory of Medical Genomics, Shanghai 200025, China

**Keywords:** 多发性骨髓瘤, 造血干细胞移植，自体, 造血干细胞动员, 普乐沙福, Multiple myeloma, Hematopoietic stem cell transplantation, autologous, Hematopoietic stem cell mobilization, Plerixafor

## Abstract

**目的:**

回顾性分析新药时代不同诱导方案对多发性骨髓瘤患者外周血自体造血干细胞动员的影响。

**方法:**

回顾性收集2022年11月至2024年11月在上海交通大学医学院附属瑞金医院进行自体造血干细胞动员的新诊断多发性骨髓瘤患者140例，根据患者的用药情况将其分为三组：以硼替佐米为基础方案（V）组37例、以硼替佐米+来那度胺为基础方案（VR）组28例、以达雷妥尤单抗+硼替佐米+来那度胺为基础方案（DVR）组75例。比较不同诱导化疗方案对造血干细胞动员、采集成功率、优良率的影响。

**结果:**

140例患者中男73例，女67例，中位年龄58（34～71）岁，V组、VR组、DVR组单次造血干细胞采集成功率分别为94.6％、82.1％、73.3％（*P*<0.05），单次造血干细胞采集优良率分别为70.3％、50.0％、36.0％（*P*<0.01）。多因素Logistic回归分析显示，来那度胺应用≥2个周期是影响造血干细胞动员采集成功率的不良因素（*OR*＝0.25，95％*CI*：0.08～0.78，*P*<0.05）；达雷妥尤单抗应用≥2个周期是影响造血干细胞动员采集优良率的不良因素（*OR*＝0.40，95％ *CI*：0.20～0.82，*P*<0.05）。动员前血小板计数≥150×10^9^/L（*OR*＝7.89，95％*CI*：2.43～25.62，*P*<0.001）、采集造血干细胞前1 d CD34^+^细胞计数≥2个/µl（*OR*＝14.85，95％*CI*：4.67～47.16，*P*<0.001）为造血干细胞采集成功的独立预测因素；动员前白细胞计数≥4.5×10^9^/L（*OR*＝2.35，95％ *CI*：1.01～5.45，*P*＝0.046）、动员前血小板计数≥150×10^9^/L（*OR*＝5.85，95％*CI*：1.72～19.94，*P*＝0.005）、采集造血干细胞前1 d CD34^+^细胞计数≥10个/µl为造血干细胞采集优良的独立预测因素（*OR*＝10.45，95％ *CI*：4.26～25.63，*P*<0.001）。根据采集造血干细胞前1 d外周血CD34^+^细胞计数及时加用普乐沙福抢救动员提高了27.8％的造血干细胞采集成功率和5.0％的优良率。

**结论:**

新药时代不同诱导化疗方案对造血干细胞的动员采集有明显影响，其中来那度胺和达雷妥尤单抗影响较大，根据采集前1 d的CD34^+^细胞计数及时予普乐沙福抢救动员可以提高造血干细胞采集的成功率和优良率。

## Abstract (en)

**Objective:**

This study aimed to retrospectively analyze the impact of different induction regimens on autologous hematopoietic stem cell mobilization in the peripheral blood of patients with multiple myeloma in the new drug era.

**Methods:**

This study retrospectively analyzed the data of 140 patients with newly diagnosed multiple myeloma who underwent autologous hematopoietic stem cell mobilization at Ruijin Hospital Affiliated to Shanghai Jiao Tong University School of Medicine from November 2022 to November 2024. The patients were categorized based on their treatment regimens into the bortezomib-based group（V, 37 cases）, bortezomib + lenalidomide-based group（VR, 28 cases）, and daratumumab + bortezomib + lenalidomide-based group（DVR, 75 cases）. The study compared the effects of different induction chemotherapy regimens in terms of hematopoietic stem cell mobilization, collection success rate, and quality rate.

**Results:**

Among the 140 patients, 73 were males and 67 were females, and the median age was 58（range, 34–71）years. The success rates of first-time hematopoietic stem cell collection were 94.6％, 82.1％, and 73.3％ in the V, VR, and DVR groups, respectively（*P*<0.05）. The quality rates of first-time hematopoietic stem cell collection were 70.3％, 50.0％, and 36.0％ in the V, VR, and DVR groups, respectively（*P*<0.01）. Multivariate logistic regression analysis revealed that lenalidomide use for ≥2 cycles was an adverse factor affecting collection success（*OR*＝0.25, 95％ *CI*: 0.08–0.78, *P*<0.05）, whereas daratumumab use for ≥2 cycles was an adverse factor for collection quality（*OR*＝0.40, 95％ *CI*: 0.20–0.82, *P*<0.05）. A premobilization platelet count ≥150×10^9^/L（*OR*＝7.89, 95％ *CI*: 2.43–25.62, *P*<0.001）and a CD34^+^ cell count of ≥2/µl on the day before collection（*OR*＝14.85, 95％ *CI*: 4.67–47.16, *P*<0.001）were considered independent predictors of successful collection. A premobilization white blood cell count of ≥4.5×10^9^/L（*OR*＝2.35, 95％ *CI*: 1.01–5.45, *P*＝0.046）, a premobilization platelet count of ≥150×10^9^/L（*OR*＝5.85, 95％ *CI*: 1.72–19.94, *P*<0.005）, and a CD34^+^ cell count of ≥10/µl on the day before collection（*OR*＝10.45, 95％ *CI*: 4.26–25.63, *P*<0.001）were considered independent predictors for collection quality. Timely administration of plerixafor based on the CD34^+^ cell count on the day before collection improved the success rate by 27.8％ and the quality rate by 5.0％.

**Conclusion:**

In the new drug era, different induction chemotherapy regimens significantly impact hematopoietic stem cell mobilization and collection, with lenalidomide and daratumumab exhibiting notable effects. Timely administration of plerixafor for salvage mobilization based on CD34^+^ cell counts on the day before collection improves both the success rate and quality rate of hematopoietic stem cell collection.

多发性骨髓瘤（MM）是一种常见的血液系统恶性肿瘤，占所有血液系统恶性肿瘤的10％[1]。近年来，以硼替佐米、来那度胺、达雷妥尤单抗为代表的新药的应用显著改善了患者的整体疗效，使MM的治疗进入了“新药时代”[2]。大量临床研究表明，自体造血干细胞移植（auto-HSCT）目前仍是适合移植的新诊断MM患者的一线治疗选择，即使在新药时代，auto-HSCT的地位仍不可替代[3]。然而，包含新药的诱导化疗方案对MM患者自体造血干细胞的动员和采集是否有影响、在造血干细胞动员过程中如何适时加用新的动员剂普乐沙福仍未明确，为解决上述问题，本研究对140例MM患者自体造血干细胞动员和采集情况进行回顾性分析。

## 病例与方法

1. 病例：收集2022年11月至2024年11月在上海交通大学医学院附属瑞金医院进行自体造血干细胞动员的140例新诊断适合移植MM患者的临床资料，所有患者的临床资料完整，符合《中国多发性骨髓瘤诊治指南（2024年修订）》[4]的诊断标准。遗传学危险分层高危定义为包含del（17p）、1q21扩增、t（4;14）、t（14;16）、t（14;20）中的1种及以上，其余为标危[4]。

2. 疗效评估：包括严格意义的完全缓解（sCR）、完全缓解（CR）、非常好的部分缓解（VGPR）、部分缓解（PR），均参考2016年国际骨髓瘤工作组（IMWG）的疗效标准[5]。

3. 化疗方案：140例患者根据新药应用情况分为三组：以硼替佐米为基础方案（V）组37例，主要包括PAD方案（硼替佐米+脂质体阿霉素+地塞米松）/PCD方案（硼替佐米+环磷酰胺+地塞米松）；以硼替佐米+来那度胺为基础方案（VR）组28例，主要包括VRD方案（硼替佐米+来那度胺+地塞米松）/VRDA方案（硼替佐米+来那度胺+地塞米松+脂质体阿霉素）；以达雷妥尤单抗+硼替佐米+来那度胺为基础方案（DVR）组75例，主要包括Dara+VRD（达雷妥尤单抗+硼替佐米+来那度胺+地塞米松）/Dara+VRDA方案（达雷妥尤单抗+硼替佐米+来那度胺+地塞米松+脂质体阿霉素）。

上述方案中硼替佐米、来那度胺和达雷妥尤单抗的常规剂量均按照药品说明书标准剂量给药（硼替佐米1.3 mg/m^2^，4或3次；来那度胺25 mg或10 mg，21 d；达雷妥尤单抗16 mg/kg，3或2次），若治疗过程中患者出现感染、白细胞减少或严重不良反应，则相应减少用药剂量或次数。中位诱导治疗4（3～7）个疗程后，若患者疗效达到PR及以上，择期行外周血造血干细胞动员和采集。

4. 外周血造血干细胞动员：本研究仅纳入首次动员患者，采用化疗动员及稳态动员方式。其中109例（77.8％）采用化疗联合粒细胞集落刺激因子（G-CSF）动员，给药方案：环磷酰胺60 mg/kg±依托泊苷6 mg/kg，分2 d给药；化疗结束1周后予G-CSF 10 µg·kg^−1^·d^−1^，连续皮下注射。当WBC从低谷回升至正常时开始采集造血干细胞（大部分为G-CSF应用第5天），采集前1 d早晨6∶00采集外周血测CD34^+^细胞数，根据CD34^+^细胞数决定是否加用普乐沙福抢救动员。另外31例（22.1％）直接采用稳态动员，即G-CSF单药或联合普乐沙福动员，给药方案：G-CSF 10 µg·kg^−1^·d^−1^，第1～5天皮下注射；动员第4天晚上23∶00皮下注射普乐沙福24 mg；第5天上午9∶00开始采集外周血造血干细胞。

5. 外周血造血干细胞采集：所有患者采用Spectra Optia血细胞分离机采集外周血造血干细胞，流式细胞术进行CD34^+^细胞计数，并根据患者体重计算采集结果。设定采集循环血量为患者血容量的2.5～3倍，统计患者首次造血干细胞采集结果，即第1天的采集量、采集成功率及优良率。根据意大利骨髓移植工作组（GITMO）标准[6]和Mayo Clinic标准[7]评估MM患者自体造血干细胞采集的效果：①采集成功：获得的CD34^+^细胞数≥2×10^6^/kg；②采集优良：获得的CD34^+^细胞数≥5×10^6^/kg；③采集失败：获得的CD34^+^细胞数<2×10^6^/kg或采集外周血造血干细胞当天因动员不佳放弃采集者。若患者第1天采集的CD34^+^细胞数<2×10^6^/kg，可行第2天采集。

6. 统计学处理：采用R 4.2软件进行统计学分析，计数资料用例数（％）描述，不符合正态分布的计量资料用中位数（范围）描述，组间数据比较采用单因素方差分析或*χ*^2^检验。采用单因素、多因素Logistic回归模型分析影响造血干细胞动员和采集的相关因素。*P*<0.05为差异有统计学意义。

## 结果

1. 临床特征：患者的基线临床特征见表1。三组患者性别、年龄、M蛋白类型、动员前WBC、动员前PLT、动员前疾病状态、动员方案等的差异均无统计学意义（*P*值均>0.05）。

**表1:** V组、VR组、DVR组多发性骨髓瘤患者的基线临床特征［例（％）］

临床特征	总体（140例）	V组（37例）	VR组（28例）	DVR组（75例）	*χ*^2^值^a^	*P*值^a^
性别					0.462	0.794
女	67（47.9）	19（51.3）	12（42.9）	36（48.0）		
男	73（52.1）	18（48.7）	16（57.1）	39（52.0）		
年龄					0.044	0.978
≤60岁	89（63.6）	23（62.2）	18（64.3）	48（64.0）		
>60岁	51（36.4）	14（37.8）	10（35.7）	27（36.0）		
M蛋白类型					6.611	0.579
IgG型	64（45.7）	16（43.2）	16（57.1）	32（42.7）		
IgA型	38（27.1）	11（29.7）	6（21.4）	21（28.0）		
IgD型	6（4.3）	1（2.7）	1（3.6）	4（5.3）		
轻链型	29（20.7）	7（18.9）	4（14.3）	18（24.0）		
不分泌型	3（2.1）	2（5.4）	1（3.6）	0（0）		
R-ISS分期					14.317	0.006
Ⅰ期	45（32.1）	17（45.9）	13（46.4）	15（20.0）		
Ⅱ期	78（55.7）	19（51.4）	13（46.4）	46（61.3）		
Ⅲ期	17（12.1）	1（2.7）	2（7.1）	14（18.7）		
遗传学危险分层					8.653	0.013
低危	72（51.4）	23（62.2）	19（67.9）	30（40.0）		
高危	68（48.6）	14（37.8）	9（32.1）	45（60.0）		
动员前WBC					2.490	0.288
<4.5×10^9^/L	54（38.8）	14（37.8）	14（51.9）	26（34.7）		
≥4.5×10^9^/L	85（61.2）	23（62.2）	13（48.1）	49（65.3）		
动员前PLT					2.480	0.289
<150×10^9^/L	25（18.0）	5（13.5）	3（11.1）	17（22.7）		
≥150×10^9^/L	114（82.0）	32（86.5）	24（88.9）	58（77.3）		
动员前疾病状态					1.272	0.973
PR	26（18.6）	7（18.9）	6（21.4）	13（17.3）		
VGPR	52（37.1）	13（35.1）	12（42.9）	27（36.0）		
CR	47（33.6）	13（35.1）	7（25.0）	27（36.0）		
sCR	15（10.7）	4（10.8）	3（10.7）	8（10.7）		
动员方案					2.497	0.287
稳态动员	31（22.1）	5（13.5）	6（21.4）	20（26.7）		
化疗动员	109（77.9）	32（86.5）	22（78.6）	55（73.3）		
化疗周期数					34.874	<0.001
≤4个	97（69.3）	36（97.3）	25（89.3）	36（48.0）		
>4个	43（30.7）	1（2.7）	3（10.7）	39（52.0）		
来那度胺周期数					90.983	<0.001
<2个	50（35.7）	37（100）	5（17.9）	8（10.7）		
≥2个	90（64.3）	0（0）	23（82.1）	67（89.3）		
达雷妥尤单抗周期数					83.325	<0.001
<2个	83（59.3）	37（100）	28（100）	18（24.0）		
≥2个	57（40.7）	0（0）	0（0）	57（76.0）		

**注** V：以硼替佐米为基础方案；VR：以硼替佐米+来那度胺为基础方案；DVR：以达雷妥尤单抗+硼替佐米+来那度胺为基础方案；PR：部分缓解；VGPR：非常好的部分缓解；CR：完全缓解；sCR：严格意义的完全缓解；^a^V组、VR组、DVR组比较

DVR组R-ISS分期Ⅱ期、Ⅲ期患者比例高，分别占61.3％、18.7％，且高危患者占60.0％，三组患者R-ISS分期和高危患者比例的差异均有统计学意义（*P*值均<0.05）。

患者中位诱导化疗4（3～7）个周期，>4个周期的患者43例，其中V组1例，VR组3例，DVR组39例，三组的差异有统计学意义（*P*<0.001）。

2. 影响造血干细胞采集的因素分析：单因素分析显示，患者的性别、年龄（>60岁/≤60岁）、疾病类型、分期（DS、ISS、R-ISS分期）、遗传学危险分层、动员前疾病状态、动员方案与第1次造血干细胞动员采集成功率及优良率均无显著相关性（*P*值均>0.05）。

（1）影响造血干细胞采集成功率的因素分析：单因素分析显示，化疗周期>4个、来那度胺应用≥2个周期、达雷妥尤单抗应用≥1个周期与造血干细胞动员采集成功率相关（*P*<0.05）；多因素分析显示，来那度胺应用≥2个周期的患者造血干细胞采集成功率更低，是造血干细胞采集失败的独立影响因素（*P*<0.05）。

（2）影响造血干细胞采集优良率的因素分析：单因素分析显示，来那度胺应用≥3个周期、达雷妥尤单抗应用≥2个周期与造血干细胞采集优良率相关（*P*<0.05）；多因素分析显示：达雷妥尤单抗应用≥2个周期的患者造血干细胞采集优良率更低，是造血干细胞采集优良的独立预后因素（*P*<0.05）（表2）。

**表2:** 影响多发性骨髓瘤患者第1次造血干细胞采集成功率及优良率的单因素、多因素分析

因素	影响采集指标	单因素分析		多因素分析	
		*OR*（95％*CI*）	*P*值	*OR*（95％*CI*）	*P*值
来那度胺应用≥2个周期	成功率	0.25（0.08～0.78）	0.017	0.25（0.08～0.78）	0.017
来那度胺应用≥3个周期	优良率	0.44（0.21～0.89）	0.023	0.50（0.24～1.05）	0.066
达雷妥尤单抗应用≥1个周期	成功率	0.33（0.13～0.85）	0.021	–	–
达雷妥尤单抗应用≥2个周期	优良率	0.36（0.18～0.74）	0.005	0.40（0.20～0.82）	0.013
化疗>4个周期	成功率	0.39（0.16～0.92）	0.032	–	–

**注** –：未进行多因素分析

3. 造血干细胞采集情况比较：140例患者共行造血干细胞采集146次。11例患者因外周血CD34^+^细胞计数太少放弃采集（V组、VR组、DVR组分别有1例、2例和8例）；17例患者因第1天CD34^+^细胞采集量<2×10^6^/kg于第2天行二次采集（V组、VR组、DVR组分别有1例、3例和13例）。

V组、VR组、DVR组患者第1次造血干细胞采集量中位数分别为7.56（0～27.80）×10^6^/kg、5.18（0～15.39）×10^6^/kg、3.55（0～22.06）×10^6^/kg，三组的差异有统计学意义（*F*值＝8.135，*P*<0.001）。V组、VR组、DVR组第1次造血干细胞采集成功率分别为94.6％、82.1％、73.3％，三组的差异有统计学意义（*χ*^2^值＝7.240，*P*＝0.027）。V组、VR组、DVR组第1次造血干细胞采集优良率分别为70.3％、50.0％和36.0％，三组的差异有统计学意义（*χ*^2^值＝11.730，*P*＝0.003）。

4. 造血干细胞采集成功率及优良率的预测因素：化疗动员组共109例，采用化疗联合G-CSF动员，其中84例（77.1％）为G-CSF应用第5天开始采集造血干细胞，25例（22.9％）因为血象恢复不佳，推迟到G-CSF应用第6天采集造血干细胞。有18例（16.5％）化疗动员患者因采集前CD34^+^细胞数未达标加用普乐沙福抢救动员，该18例患者经抢救动员后第1次采集的造血干细胞平均值为6.99×10^6^/kg。稳态动员组共31例，其中26例（83.9％）于动员第5天开始采集外周血干细胞，5例（16.1％）因CD34^+^细胞数上升不佳，G-CSF皮下注射增加1 d，推迟到动员第6天采集外周血干细胞。稳态动员组31例患者中有21例（67.7％）应用了普乐沙福，这21例患者第1次采集的造血干细胞平均值为4.46×10^6^/kg。

单因素分析显示，动员前WBC≥4.5×10^9^/L、动员前PLT≥150×10^9^/L、采集造血干细胞前1 d CD34^+^细胞计数≥2个/µl均与造血干细胞采集成功率相关（*P*值均<0.05）；动员前WBC≥4.5×10^9^/L、动员前PLT≥150×10^9^/L、采集造血干细胞前1 d CD34^+^细胞计数≥10个/µl均与造血干细胞采集优良率相关（*P*值均<0.05）。

多因素分析显示，动员前PLT≥150×10^9^/L、采集造血干细胞前1 d CD34^+^细胞计数≥2个/µl均为造血干细胞采集成功的独立预测因素（*P*值均<0.001）；动员前WBC≥4.5×10^9^/L、动员前PLT≥150×10^9^/L、采集造血干细胞前1 d CD34^+^细胞计数≥10个/µl均为造血干细胞采集优良的独立预测因素（*P*值均<0.05）。

采集造血干细胞前1 d CD34^+^细胞计数≥2个/µl与单次采集造血干细胞成功率的相关性更高（*OR*＝14.85，*P*<0.001），采集造血干细胞前1 d CD34^+^细胞计数≥10个/µl与单次采集造血干细胞优良率的相关性更高（*OR*＝10.45，*P*<0.001），可作为造血干细胞采集成功及优良的预测因素（表3）。

**表3:** 预测多发性骨髓瘤患者单次造血干细胞采集成功率、优良率的单因素、多因素分析

因素	影响采集指标	单因素分析		多因素分析	
		*OR*（95％*CI*）	*P*值	*OR*（95％*CI*）	*P*值
动员前WBC≥4.5×10^9^/L	成功率	3.45（1.44～8.26）	0.006	3.27（1.14～9.35）	0.147
	优良率	2.59（1.28～5.28）	0.008	2.35（1.01～5.45）	0.046
动员前PLT≥150×10^9^/L	成功率	6.09（2.35～15.82）	<0.001	7.89（2.43～25.62）	<0.001
	优良率	4.60（1.62～13.11）	0.004	5.85（1.72～19.94）	0.005
采集造血干细胞前1 d CD34^+^细胞≥2个/µl[8]–[9]	成功率	12.87（4.74～34.96）	<0.001	14.85（4.67～47.16）	<0.001
采集造血干细胞前1 d CD34^+^细胞≥10个/µl[8]–[9]	优良率	8.54（3.87～18.87）	<0.001	10.45（4.26～25.63）	<0.001

140例患者中113例（80.7％）造血干细胞采集成功，27例（19.3％）采集失败。多因素分析结果显示，采集造血干细胞前1 d CD34^+^细胞计数<2个/µl的患者采集成功率为40.0％，≥2个/µl的患者采集成功率为89.6％，差异有统计学意义（*P*<0.001）。140例患者中67例（47.9％）采集优良，73例（52.1％）未达优良，采集造血干细胞前1 d CD34^+^细胞计数<10个/µl的患者采集优良率为29.1％，≥10个/µl的患者采集优良率为77.8％，差异有统计学意义（*P*<0.001）。

5. 普乐沙福对造血干细胞采集成功率及优良率的影响：DVR组采集造血干细胞前1 d CD34^+^细胞计数<2个/µl患者17例，其中9例未加用普乐沙福，单次造血干细胞采集成功率为22.2％；另外8例患者加用普乐沙福，单次采集造血干细胞成功率为50.0％，普乐沙福使造血干细胞采集成功率提升了27.8％。DVR组采集造血干细胞前1 d CD34^+^细胞计数<10个/µl患者50例，其中31例患者继续单用G-CSF动员，造血干细胞采集优良率为16.1％；其余19例患者加用普乐沙福，造血干细胞采集优良率为21.1％，普乐沙福使造血干细胞采集优良率提升了5.0％。

## 讨论

在新药时代，硼替佐米、来那度胺、达雷妥尤单抗已成为MM标准治疗的常用药物，显著改善了患者的疗效，明显延长了患者的生存期。然而，新型药物的加入也对造血干细胞的动员和采集产生了影响。本研究显示，含有来那度胺、达雷妥尤单抗的诱导化疗会显著影响造血干细胞的动员。

来那度胺是一种免疫调节药物。一项研究表明，动员前接受3个或以上周期来那度胺治疗患者的造血干细胞采集失败风险较高，原因可能与来那度胺上调造血干细胞表面的CXCR4受体导致造血干细胞滞留在骨髓造血干细胞龛中有关[10]–[11]。因此，添加CXCR4拮抗剂（普乐沙福）已被证明可以减轻来那度胺对造血干细胞动员的不良影响[12]–[13]。动员前限制来那度胺的应用周期数可以改善动员效率[13]–[14]，与本研究结论相似。VR组第1次造血干细胞采集量、采集成功率、采集优良率均明显低于V组，为了提高第1次造血干细胞采集的成功率和优良率，建议动员前将来那度胺的应用限制在2个周期以内，若超出此限制，则需考虑多次采集或加用普乐沙福。

达雷妥尤单抗是一种靶向CD38的单克隆抗体，已越来越多地纳入MM的一线治疗方案中。研究表明，应用达雷妥尤单抗可能会对动员的造血干细胞的数量和质量产生负面影响。一项比较含达雷妥尤单抗方案（Dara-VRD）与不含达雷妥尤单抗方案（VRD）疗效的研究显示，两组患者造血干细胞采集成功率分别为44％和71％，表明应用达雷妥尤单抗可能会影响造血干细胞动员[15]，其机制可能与MM细胞和CD34^+^细胞上CD38的表达有关，虽然达雷妥尤单抗有效靶向MM细胞上的CD38，也可能影响表达较低水平CD38的CD34^+^细胞。达雷妥尤单抗与CD38的结合可能会干扰CD34^+^细胞从骨髓流向外周血，导致CD34^+^细胞动员减少[15]–[16]。接受含达雷妥尤单抗诱导治疗的患者对普乐沙福的依赖性更高[17]–[18]。本研究结果同样显示，达雷妥尤单抗会影响造血干细胞的动员，DVR组第1次造血干细胞采集量、采集成功率、采集优良率均明显低于VR组，DVR组放弃采集造血干细胞或行二次采集的患者比例均明显高于VR组。为了提高造血干细胞的动员效率，建议在采集造血干细胞前将达雷妥尤单抗的应用限制在2个周期以内，如因病情需增加用药周期，应密切监测外周血CD34^+^细胞数，及时加用普乐沙福，以提高造血干细胞采集的成功率和优良率。

硼替佐米是一种蛋白酶体抑制剂，有研究表明，硼替佐米的应用不会对造血干细胞的动员和采集产生负面影响[19]–[20]。也有研究显示，硼替佐米与G-CSF联合应用可以增强造血干细胞的动员能力[21]。本研究结果也显示，V组造血干细胞动员和采集基本不受影响，单次造血干细胞的采集量、采集成功率和优良率均明显优于VR组和DVR组，与上述研究结论一致。

在新药时代，诱导方案对MM患者造血干细胞的动员和采集产生了重要的影响。本研究显示，化疗动员或稳态动员均不影响造血干细胞采集的成功率及优良率。然而，化疗动员组109例患者中18例（16.5％）应用了普乐沙福，第1次造血干细胞采集的平均值为6.99×10^6^/kg，稳态动员组31例患者21例（67.7％）应用了普乐沙福，第1次造血干细胞采集的平均值为4.46×10^6^/kg，化疗动员组高于稳态动员组。采集量的增加对于需要行双次串联移植的患者尤为重要，更高的造血干细胞数量和更短的植入时间与患者的预后相关，减少采集次数能够减轻患者的负担[22]。化疗动员并非没有缺点，尽管化疗动员可以提高采集量，但化疗带来的不良反应增加，包括血液学不良反应和感染风险等[19],[23]。

有研究表明，动员前WBC<4.0×10^9^/L、PLT<150×10^9^/L可能是造血干细胞动员不佳的危险因素[24]–[26]。本研究也得出了类似结论，动员前WBC≥4.5×10^9^/L、动员前PLT≥150×10^9^/L与造血干细胞采集成功率和优良率均呈正相关，可作为患者采集造血干细胞前的预测因素。采集造血干细胞前1 d CD34^+^细胞计数是造血干细胞采集成功率和优良率更精准的预测因素。有研究显示，采集造血干细胞前1 d CD34^+^细胞计数≥2个/µl表明造血干细胞的动员尚可，与采集到的CD34^+^细胞数量密切相关。CD34^+^细胞计数≥10个/µl被认为是造血干细胞动员效果好的临界值，通常在采集过程中可获得足够数量的CD34^+^细胞[8]–[9]。采集造血干细胞前1 d CD34^+^细胞计数≥10个/µl可作为采集优良的早期预测指标，可以使临床医师更好地安排采集时机，从而获得更好的采集结果[27]–[28]，本研究结果也证实了这一点。普乐沙福是一种新型造血干细胞动员剂，稳态动员或化疗动员中加入普乐沙福均可使造血干细胞采集量明显增加[29]–[30]。在患者造血干细胞动员过程中动态监测CD34^+^细胞计数，并根据采集造血干细胞前1 d CD34^+^细胞计数适时加用普乐沙福进行挽救性动员可以提高造血干细胞采集的成功率和优良率[13]。本研究结果提示，采集造血干细胞前1 d CD34^+^细胞计数<2个/µl患者应考虑加用普乐沙福提高采集成功率。若想提高动员效果，建议采集前1 d CD34^+^细胞计数<10个/µl的患者加用普乐沙福。
